# Strategies and recommendations for the management of gastrointestinal surgery during the COVID-19 pandemic: experience shared by Chinese surgeons

**DOI:** 10.1093/gastro/goaa030

**Published:** 2020-07-03

**Authors:** Jia Ke, Nan Lan, Ting Wang, Jin-Jie Wu, Zhen He, Xiao-Sheng He, Kai-Xiong Tao, Qun Qian, Ping-Hong Zhou, Guo-Xin Li, Min-Hua Zheng, Zhong-Tao Zhang, Jia-Fu Ji, Ping Lan

**Affiliations:** g1 Department of Colorectal Surgery, The Sixth Affiliated Hospital, Sun Yat-sen University, Guangzhou, Guangdong, P. R. China; g2 Guangdong Institute of Gastroenterology, Guangdong Provincial Key Laboratory of Colorectal and Pelvic Floor Diseases, Guangzhou, Guangdong, P. R. China; g3 Department of Gastrointestinal Surgery, Union Hospital, Tongji Medical College, Huazhong University of Science and Technology, Wuhan, Hubei, P. R. China; g4 Department of General Surgery, Zhongnan Hospital of Wuhan University, Wuhan, Hubei, P. R. China; g5 Endoscopy Center, Zhongshan Hospital, Fudan Univeristy, Shanghai, P. R. China; g6 Department of General Surgery, Nanfang Hospital, Southern Medical University, Guangzhou, Guangdong, P. R. China; g7 Department of Gastrointestinal Surgery, Ruijin Hospital, Shanghai Jiaotong University School of Medicine, Shanghai, P. R. China; g8 Department of General Surgery, Beijing Friendship Hospital, Capital Medical University, National Clinical Research Center for Digestive Diseases, Beijing, P. R. China; g9 Department of Gastrointestinal Cancer Center, Key Laboratory of Carcinogenesis and Translational Research (Ministry of Education), Peking University Cancer Hospital & Institute, Beijing, P. R. China

**Keywords:** gastroenterological surgery, novel coronavirus disease-2019, perioperative care, severe acute respiratory syndrome coronavirus 2

## Abstract

Novel coronavirus disease-2019 (COVID-19), caused by severe acute respiratory syndrome coronavirus 2 (SARS-CoV-2), is an ongoing public-health pandemic worldwide. Although SARS-CoV-2 has been known to spread primarily through respiratory droplets, recent evidence also supports fecal/oral as an additional route of transmission, raising concerns over gastrointestinal (GI) transmission of the infection. Herein, we, as the front-line Chinese GI surgeons, would like to share our experience and lessons in the combat against COVID-19. It is essential to create science-based, rational, and practical strategies during the outbreak of COVID-19. Here, we provide multi-institutional consensus on minimizing disease transmission while continuing to provide care from all aspects for patients in GI surgery, including outpatient clinics, inpatient units, gastrointestinal endoscopy centers, and adjustments in perioperative care. Our experiences and recommendations are worth sharing and may help to establish specific infection-control and outcome measures.

## Introduction

The novel coronavirus disease-2019 (COVID-19) pandemic is caused by an enveloped, single-stranded RNA virus, named severe acute respiratory syndrome coronavirus 2 (SARS-CoV-2) [1, 2]. The ongoing COVID-19 pandemic continues to be a major issue with the increasing number of confirmed cases and disease-related fatalities worldwide. Although SARS-CoV-2 spreads primarily through respiratory droplets, recent evidence suggests the fecal/oral route as another potential means of transmission, raising concerns over gastrointestinal (GI) manifestations of the disease [3, 4]. Since multiple GI disorders present with fever, distinguishing between COVID-19-related symptoms and other diseases can be challenging. In addition, asymptomatic carriers and patients with absence of or minimal symptoms during the incubation period also make diagnosis difficult and therefore play an important part in expediting the outbreak. Therefore, the decision on when to proceed with surgical procedures and when can we safely delay procedures became one of the major challenges in this pandemic. Risk and benefit should be carefully considered and weighed.

The aim of this consensus is to share multicenter experiences from front-line GI surgeons regarding: (i) minimizing SARS-CoV-2 transmission, (ii) adjustments for diagnostic and therapeutic GI procedures in all units of the department, and (iii) improvements in perioperative preventive care.

## Overall strategies for infection prevention and control

Overall strategies play a principle role in the guidance of SARS-CoV-2-infection prevention and control during the outbreak of COVID-19 [5]. They are described as follows.

### Establishing a task force

We recommend that each hospital establishes a COVID-19 task force as early as possible. The responsibilities of the task force include, but are not limited to, making in-house guidelines; organizing multidisciplinary treatment (MDT); and managing, coordinating, and implementing procedures according to preventive precautions. Most importantly, the task force should have overall control regarding the balance between the resources being used for COVID-19 patients (e.g. personal protection equipment [PPE], ventilators, intensive care unit [ICU] beds, physicians) and those that would be used for surgical patients.

We also recommend that each hospital should establish a group of diagnostic experts with responsibilities for risk stratification, especially for patients under investigation who need urgent surgery.

### Risk stratification of patients and corresponding responses

In an effort to identify the risk and to guide proper triage, patients are classified into the following three categories (Table 1) [6].

**Table 1.** Hierarchical precautions corresponding to the risk classification of patients and tasks of medical personnel

Levels of precautions	Personal protective equipment	Risk classification of patients	Medical personnel to be applied in areas
General/standard	Hand hygiene; Surgical mask; Medical cap; Disposable medical gloves; Medical clothes	Patients at low risk	Inpatient-ward clean-area personnel
Level One^a^	Hand hygiene; Surgical mask or N95 mask; Medical cap; Medical clothes; Disposable medical gloves; Medical shoe covers; Single-use isolation gown	Patients under investigation	Outpatient clinics; Operating room personnel; Inpatient-ward non-clean-area personnel
Level Two^a^	Hand hygiene; Medical N95 respirator; Goggles; Medical clothes; Medical gloves; Protective clothing; Medical cap; Medical shoe covers	Patients at high risk/with confirmed COVID-19	Fever clinics, performing diagnosis and treatment, nursing, cleaning, and transportation for suspected patients; Endoscopic unit personnel
Level Three	Hand hygiene; Medical N95 respirator; Comprehensive respirator or positive-airway press headgear; Protective clothing; Medical cap; Medical shoe covers	Patients with confirmed COVID-19	Performing procedures with possible production of aerosol sprays in suspected or confirmed patients (endotracheal intubation, tracheal suction, endoscopic treatments, etc.)

a If possible, the level of precautions could be elevated in the pandemic area.

#### Patients with confirmed COVID or patient at high risk

For a patient with GI disease also infected by SARS-CoV-2 or at high risk for COVID-19, he/she must be transferred to an isolation ward in the department of infectious disease, jointly treated by infectious-disease specialists and surgeons. If possible, single-person, single-room, and Level Two and Level Three protection should take place. Clear notification, which we proposed should be in red, must be posted on the door to raise caution.

#### Patients at low risk

Patients without close contact with confirmed COVID-19 cases or not coming from a high-risk environment in the past 14 days in the absence of fever, respiratory symptoms, and radiological findings are classified as low-risk. A notification sign in green should be posted on the door of the ward. If there is a shortage of rooms, patients in this category can be placed in the same room at a distance of 1.5–2.0 meters between beds with general/standard precautions.

#### Patients under investigation

The remaining patients should be considered patients under investigation (PUIs). They can be further classified into the following two subgroups:

- patients who presented with some symptoms of respiratory-tract infection but do not fulfill the criteria for COVID-19;
- patients in need of urgent or emergent care who present without signs of fever or pulmonary involvement or patients with undetermined potential exposure; patients in this category should be cared for in a single room with a yellow sign placed on the door and Level One protection should be implemented.

#### Adjusting patient care to the risk stratification

- We recommend the following responses in correspondence to the risk stratification. Patient rounds should be conducted in the following order: patients at low risk, PUIs, and patients confirmed or at high risk. Before entering and exiting a patient’s room, and upon completion of patient rounds, gloves along with other forms of PPE should be removed and properly disposed of, immediately followed by hand-hygiene procedures.
- With the disease progression and improvement in COVID-19-detection methods, the risk stratification and corresponding protection levels should be updated dynamically. Once the patient’s status is updated as COVID-19-positive or PUI from low-risk, the cases must be reported, and the risk classification as well as the corresponding levels of prevention and protection adjusted accordingly as soon as possible.
- The working space should be divided into a clean area (e.g. work rooms, on-call room, locker rooms), a mixed or transitional area (other than clean and contaminated areas), and a contaminated area (e.g. wards for confirmed or patients at high risk). Entry to the clean, transitional, and contaminated areas should be allowed for medical personnel and only on an as-needed basis. Patients and visitors should not be allowed into designated clean areas. All personnel should change and remove any exposed clothing before entering clean areas, while personnel who wear casual clothing should not be allowed to enter the transitional area and contaminated area.
- Patient and family education measures are strongly recommended to raise awareness and to facilitate further screening. No visitation policy should be instituted. Exceptions can be made on a case-by-case basis if family a member or personal caregiver needs to be present. This should be limited to one person and he/she needs to be ruled out of having COVID-19.

## Recommendations for infection prevention in the department of GI surgery

### Ambulatory care

In general, Level One precautions should be implemented in the outpatient setting. In addition, we recommend the following [7, 8].

#### Differential diagnosis on fever patterns

- It is known that fever is one of the most common symptoms of COVID-19 and that patients with certain GI diseases (e.g. acute appendicitis, gastric perforation, intestinal obstruction) who required urgent care with emergency GI surgery often present with high fever as well. It is of critical importance to make a differential diagnosis [7].
- Patients with COVID-19 frequently have persistent mild to moderate fever accompanied by respiratory symptoms (e.g. cough, shortness of breath).
- Fever also occurs in patients with GI cancers, which is characterized by undulant fever that is different from the pattern of continuous fever in patients with COVID-19. In particular, GI-cancer patients receiving chemotherapy or complicated with other chronic diseases such as diabetes are highly susceptible to COVID-19 mainly due to immunosuppression.
- The infection often has atypical manifestations. Patients with active inflammatory bowel disease (IBD) may develop fever. When the fever is >38°C and persistent, patients should undergo expedient testing for COVID-19 [8]. IBD patients often require immunomodulators or immunosuppressive drugs, which might expose them to a potentially increased risk of SARS-CoV-2 infection. It is prudent to advise these IBD patients who are living in a hot zone to adopt enhanced protective measures such as face masks, social distancing, frequent handwashing, and disinfection with hydroalcoholic gel, while maintaining their usual treatment.

#### Recommendations on physical examinations

SARS-CoV-2 RNA was reported to be detected in fecal specimens from patients with confirmed COVID-19. Digital rectal examination and anoscopy are important steps of proctology examination, but its use should be limited under the current situation. Level Two precautions are taken while performing digital rectal examination or anoscopy.

### Inpatient unit

In addition to the risk stratification of patients, the following specific recommendations are made in the inpatient setting.

#### Recommendations on the management of patients with drainage tubes

A proportion of patients on the GI-surgery ward may require the use of nasogastric drainage or feeding tubes and so they cannot wear face masks properly. This increases possibility of virus exposure and health workers should be aware and take necessary precautions. During the process of the insertion and removal of the tubes as well as catheters (e.g. nasoenteral tube, transanal drainage tube, Foley catheter) and the emptying and changing of stoma appliances, we recommend taking Level Three precautions for confirmed or high-risk patients and a minimum of Level Two precautions for PUI and low-risk patients.

#### Recommendations on the management of patients with diarrhea and vomiting

Diarrhea and vomiting are common in the GI-surgery department and might be the source of transmission. A disinfectant with chlorine (5,000–10,000 mg/L) is recommended for a small amount of waste. For a large amount of waste, it should be fully covered with bleach powder or disinfectant powder containing water-absorbing ingredients or completely covered with single-use water-absorbing materials with a sufficient amount of chlorine-containing disinfectant (5,000–10,000 mg/L) and cleaned up after ≥30 minutes of disinfection.

### GI-endoscopy unit

Diagnostic and therapeutic GI endoscopies are considered high-risk procedures during the COVID-19 outbreak. To minimize hospital-acquired infection, we recommend that GI endoscopies are limited to those procedures with the best estimated mortality/prognostic benefits [9].

#### Urgent GI endoscopy

In the setting of life-threatening GI diseases, such as acute GI bleeding, obstruction, choledocholithiasis with acute cholangitis, and biliary pancreatitis, when diagnostic and therapeutic endoscopy is required, GI endoscopy should be promptly conducted after the necessary preventive precautions have been taken. COVID-19-positive patients with GI bleeding with hemodynamic stability should undergo conservative treatments first, including angioembolization, before endoscopic treatment due to the high risk of endoscopy being an aerosol-generating procedure. The requirement for such procedures should be risk-assessed and deferred or delayed if possible.

#### Other GI endoscopies

GI endoscopies for the diagnosis or treatment of previously identified malignant polyps, acute enteritis, endoscopic biopsy of GI cancers and nutrition-tube placement should be subjects to be discussed by the MDT team. Assessment of the risks and benefits of proceeding vs delaying the procedure should be discussed.

#### Risk stratification and triage of patients requiring GI endoscopy

For confirmed/high-risk COVID-19 patients and PUIs, diagnostic and therapeutic GI endoscopies should be performed in a negative-pressure room with Level Three precautions. For low-risk patients, diagnostic and therapeutic GI endoscopies should be conducted in a regular room with Level Two precautions.

## Recommendations on GI surgeries during the outbreak of COVID-19

GI surgeries on SARS-CoV-2-positive patients increase the risk of transmission to the surgical and perioperative team. High perioperative mortality has been observed in COVID-positive patients [10].

### GI surgery in confirmed or high-risk COVID-19 patients

Elective surgeries in confirmed or high-risk COVID-19 patients should be postponed. Urgent GI surgeries should be performed under severe, life-threatening conditions like intestinal perforation, acute obstruction with peritonitis, and ongoing bleeding with hemodynamic instability [11]. An emergency plan from the MDT team should be made and Level Three precautions should be taken.

### GI surgery in PUIs for COVID-19

For patients who present with certain symptoms of COVID-19 but do not fulfill all the criteria, GI surgeries should be postponed until proper COVID 19 testing has been performed. The date and time of GI surgeries shall be determined after the diagnosis of COVID-19 has been finalized or, alternatively, when the patient is classified as low-risk by the group of diagnostic experts. However, emergency surgical management should not be delayed by pending test results, with the rationale that all patients during the pandemic should undergo COVID-19 testing and precautions taken accordingly. Surgeries for PUIs should be performed in a negative-pressure operating room with Level Three precautions.

### GI surgery in patients at low risk for COVID-19

#### GI tumors

The decision to proceed with surgery depends on both the type and the nature of the tumor, the consequences for potential delay, as well as the sufficiency of medical supplies in relation to the number of confirmed COVID-19 cases in the hospital.

*For hospitals with a relatively small number of COVID-19 patients and readily available mechanical ventilators and ICU beds*

Cases that need to be done as soon as possible [12]:

- obstructing GI cancer without improvement after endoscopic stent placement;
- locally advanced or late-stage GI cancer, with poor response to preoperative chemotherapy/chemoradiation;
- advanced GI cancer complicated by bleeding, perforation, or other severe side effects.

Cases that could be postponed [13–15]:

- malignant polyps, either with or without prior endoscopic resection;
- early-stage gastric cancer (anti-Helicobacter pylori treatment recommended);
- large, benign-appearing asymptomatic polyps.

Surgeries to be delayed with alternative treatment [16–19]:

- locally advanced gastric cancer, colon cancer, and rectal cancer can be delayed by neoadjuvant treatments;
- for a gastric or esophageal gastric-junction tumor with obstruction, a gastric tube should be placed for decompression and a gastroenteral tube can be placed for enteral nutrition support; preoperative chemotherapy should be initiated afterwards;
- endoscopic stent should be considered for obstructing colorectal cancer for symptomatic relief and neoadjuvant treatment initiated;
- for patients who have undergone neoadjuvant treatments, a ‘wait and watch’ strategy could be applied to those who have achieved total regression, with the patients’ consent; surgery or full-scale neoadjuvant treatment could be considered for those with partial regression; surgery is needed if the tumor has progressed.

*For hospitals with a relatively large number of COVID-19 patients, with a shortage of mechanical ventilators and ICU beds*

Cases that need to be done as soon as possible:

- obstructing GI cancer without improvement after endoscopic stent placement;
- GI cancer with severe bleeding or perforation.

Cases that could be postponed:

- all colorectal surgical procedures or those referred to a hospital with fewer COVID-19 patients.

Surgeries to be delayed with alternative treatment:

- refer to hospitals with fewer COVID-19 patients.

*For designated hospitals for COVID-19 patients, with no available mechanical ventilators or ICU beds*

- stop the admission of new GI patients at low risk for COVID-19;
- urgent surgery should be considered for existing hospitalized patients in the presence of perforation, obstruction, or severe bleeding;
- after recovery from COVID-19, GI-cancer patients should be referred to a hospital with fewer COVID-19 patients.

#### Surgeries for IBD patients

In general, a majority of IBD surgeries could be postponed by using alternative medications, with cautious observation to avoid potential exacerbation [20].

IBD patients in need of surgery:

- Crohn’s disease (CD) patients with persistent bleeding, perforation, diffuse peritonitis;
- CD patients with complete bowel obstruction;
- acute severe ulcerative colitis patients in need of subtotal colectomy.

Consider alternative therapies to postpone surgery:

- CD presenting with perianal abscess can be treated with incision and drainage in outpatient settings with Level One or more precautions, with seton placement after the pandemic;
- CD presenting with intra-abdominal abscess can be treated with aggressive antibiotics, imaging-guided percutaneous abscess drainage, as well as enteral nutritional support;
- CD with partial obstruction could be given total or partial enteral nutritional therapy.

#### Surgical procedures for other GI diseases

For GI benign diseases, including appendicitis, diverticulitis, intestinal ischemia, and intestinal obstruction, we recommend that conservative non-surgical management be considered first, along with puncture drainage. When patients show poor responses to non-surgical treatment, surgery can be performed [21]. As recommended for appendicitis, details of the conservative management are as follows:

- Antibiotics alone may be sufficient for simple appendicitis and therefore intravenous-antibiotics therapy is recommended for empirical treatment and can subsequently be de-escalated to oral antibiotics.
- For appendicitis patients complicated by phlegmon, empirical intravenous-antibiotics therapy is recommended with subsequent de-escalation to oral antibiotics.
- In appendicitis complicated by abscess, imaging-guided percutaneous drainage is recommended, followed by intravenous-antibiotics therapy and subsequently oral antibiotics.
- For appendicitis in the presence of perforation, we recommend empirical intravenous antibiotics; with the occurrence of peritonitis, surgery should be considered.

#### Benign anorectal diseases

- In general, all outpatient proctology clinics and procedures, with the exception of oncological and urgent cases, should be postponed/rescheduled.
- Anorectal emergencies such as thrombosed external hemorrhoids, strangulated or bleeding hemorrhoids, anorectal abscesses, anal fissures, and Fournier’s gangrene should first be conservatively managed. Conservative measures are effective for most thrombosed hemorrhoids and anal fissures, whereas a surgical drain should be timely applied to anorectal abscesses. Fournier’s gangrene has a low incidence, but a high mortality rate of 25%–73% [22], thereby requiring immediate diagnosis and surgical intervention. In the case of proctology procedures during the pandemic, outpatient procedures and local anesthesia should be favored.

## Recommendations on perioperative infection prevention

**Figure 1.**
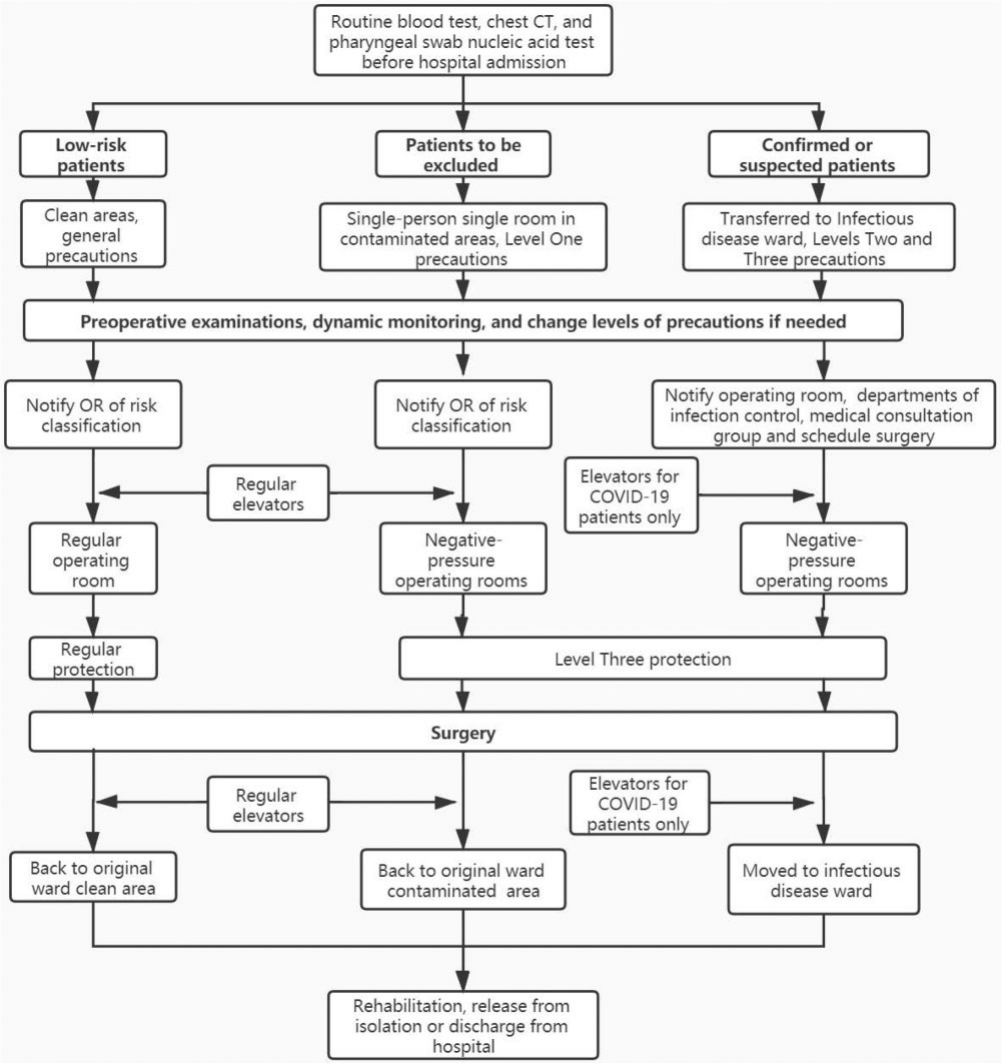
Flowchart of patient management in the gastrointestinal-surgery department according to the risk level

For all surgical personnel involved in GI surgery for confirmed/high-risk COVID-19 patients or for PUIs for COVID-19, we recommend the following protective measures (Figure 1).

### Intraoperative protective measures

#### Anesthesia process

In general, we recommend that the anesthesiologist and personnel involved in the anesthesia process should minimize aerosol production especially during the intubation process. More detailed measures in relation to the management of the anesthesia department during the COVID-19 crisis are described elsewhere [23].

#### Minimizing pathogen exposure

To minimize the exposure of the virus, we recommend the following:

- The surgeon and participating staff should pay specific attention to the scope of the operation, shorten the surgery duration whenever possible, and avoid exploring unconventional or rarely used operative procedures.
- During surgery for confirmed/high-risk COVID-19 patients or PUIs, pathogen exposure should be minimized and the surgery should be performed via an open approach; for low-risk patients, a laparoscopic approach could be considered, at the operating surgeon’s discretion.
- Caution needs to be taken when performing GI anastomosis in confirmed or high-risk COVID-19 patients in the emergency setting, because of both the high risk of complications (e.g. anastomotic leaks, intra-abdominal collections) and the subsequent consumption of healthcare resources following an anastomotic leak, whereas, in low-risk patients, protective diversion needs to be avoided, according to the surgeon’s best judgment, to minimize the risk of pathogen exposure when changing stoma appliances afterwards.
- When establishing a pneumoperitoneum in laparoscopic surgery, the puncture sites should be cleaned immediately to prevent the droplets from spreading by leakage of the air mixed with blood around the Trocar.
- Prior to taking specimens, we recommend that negative-pressure suction should be used to remove all of the aerosol in the abdominal cavity to avoid ventilating directly through the Trocar or the incision; the Trocar valve can be connected to the negative-pressure suction, which should be equipped with an appropriate filtering device to remove possible pathogens.
- The power levels of the electrosurgical or ultrasonic knife should be reduced and activating the energy device for a prolonged period of time should be avoided, to decrease smoke production; suction should be utilized as much as possible.
- We recommend using staplers for GI anastomosis.
- Certain procedures taking the transanal or transperineal approach, such as transanal total mesorectal excision (taTME) and natural orifice specimen extraction surgery, should be avoided to reduce the risk of aerosol sprays from the rectum.
- To minimize the risk of pathogen exposure, the number of tubes and drains being inserted, such as gastric tubes, intestinal tubes, transanal drainage tube, etc., should be reduced as much as possible.
- While not affecting the surgeons’ view during laparoscopic surgery, the pneumoperitoneum pressure as well as the CO2 flow rate should be reduced; the duration of the Trendelenburg’s position should be shortened to avoid the adverse effect on the lung function.
- No surgical specimen collection should be allowed for research purposes unless it is specifically related to coronavirus research.

### Post-operative protective measures

#### PUIs for COVID-19

- Patients are moved back to the contaminated area in the general ward.
- Early mobilization should be encouraged in the patient’s room to accelerate post-operative rehabilitation. In the public areas of the ward, patients should wear face masks.
- In the presence of cough and fever, the patient should be isolated according to the standard protocol of suspected COVID-19 cases, during which a chest computed tomography (CT) scan and SARS-CoV-2 RNA test should be performed.
- Careful consideration should be taken to differentiate between COVID-19 and procedure-related fever including stress response to surgery, surgical-site infection, and other procedure-related complications.

#### Suspected or confirmed patients for COVID-19

- Patients are moved to a single room within the infectious-disease ward for isolation and managed by an infectious-disease specialist together with surgeons.
- Considering that COVID-19 may progress to multi-organ dysfunction, isolation and treatments are usually prolonged; therefore, extra precautions should be taken to prevent deep venous thrombosis and hypostatic pneumonia.
- Nutritional therapy is part of the key element in the treatments for COVID-19. Post-operative enteral nutrition is recommended, supplemented by parenteral nutrition and other supplementations (e.g. fish-oil supplementation of omega-3 fatty acids, glutamine) [24, 25].

### Criteria for removing patients from isolation after surgery

We recommend meeting all the following criteria before considering removing the patient from isolation after surgery:

- body temperature returns to normal and remains afebrile for ≥3 days;
- respiratory symptoms are significantly improved;
- radiological imaging has shown significant improvement in pulmonary infiltration;
- SARS-CoV-2 RNA test is negative for two consecutive samples (sampling intervals ≥24 hours).

If the patient fulfills all the above criteria, they can be released from isolation and transferred to the general ward or discharged from hospital.

### Post-discharge follow-up visits

After being discharged from hospital, patients should be isolated at home for 14 days during which the body temperature is monitored and recorded daily. In general, as long as the prognosis has not been affected, follow-up visits can be postponed accordingly. We also recommend online/telehealth appointments and appropriate outpatient-clinic visits at locations closest to the patient.

### Adjuvant chemotherapy after resection for GI cancer

If needed, adjuvant therapy should be started 4 weeks after surgery, but no later than 8 weeks. If intravenous chemotherapy is not available due to the COVID-19 pandemic, oral capecitabine for colorectal cancer and tegafur for gastric-cancer monotherapy could be considered. The therapy should be switched to combination treatment after the pandemic is under control.

## Conclusions

During the times of the COVID-19 crisis, it is essential to establish science-based, rational, and practical strategies to ensure the safety and benefit of both caregivers and patients. Considering that the objective and subjective conditions may vary between hospitals, the combating strategies should be individualized. Our experience and recommendations are worth sharing and may help to minimize infection.

## Authors’ contributions

JK, NL, TW, JJW, ZH and XSH involved in acquisition of data, analysis of data, and drafting of the manuscript. KXT, QQ, PHZ, GXL, MHZ, ZTZ, JFJ and PL involved in study design and critical revision of the manuscript. All authors read and approved the final manuscript.

## Funding

None.
